# Supplementary material for: Investigation of Morphological, Optical, and Dielectric Properties of RF Sputtered WOx Thin Films for Optoelectronic Applications
Source: Nanomaterials (Basel). 2022 Oct 4;12(19):3467. doi: 10.3390/nano12193467 (PMC9565653; doi:10.3390/nano12193467)
Supplement: Supplementary file 1 [file nanomaterials-12-03467-s001.zip › nanomaterials-1889453-supplementary.pdf]

nanomaterials

Supplementary Material

Investigation of Morphological, Optical, and Dielectric Properties of RF Sputtered WO_x Thin Films for Optoelectronic Applications

Samiya Mahjabin [1], Md. Mahfuzul Haque [1], K. Sobayel [1], Vidhya Selvanathan [1], M. S. Jamal [2], M. S. Bashar [2,*], Munira Sultana [2], Mohammad Ismail Hossain [3], Md. Shahiduzzaman [4], Merfat Algethami [5], Sami S. Alharthi[5], Nowshad Amin [6], Kamaruzzaman Sopian [1], Md. Akhtaruzzaman [1,7,*]

1 Solar Energy Research Institute (SERI), Universiti Kebangsaan Malaysia (@The National University of Malaysia), 43600 Bangi, Malaysia; smmahjabin@gmail.com (S.M.); mahfuz.ap@gmail.com (M.M.H.); sobayel@ukm.edu.my (K. Sobayel); vidhya@ukm.edu.my (V.S.); ksopian@ukm.edu.my (Kamaruzzaman Sopian)
2 Bangladesh Council of Scientific and Industrial Research, Dhaka-1205, Bangladesh; msjamal@bcsir.gov.bd (M.S.J.), muniraeva37@gmail.com (M.S.)
3 Department of Electrical and Computer Engineering, University of California, Davis (UCD), USA; m.hossain.jub@gmail.com
4 Nanomaterials Research Institute (NanoMaRi), Kanazawa University, Kakuma, Kanazawa 920-1192, Japan; shahiduzzaman@se.kanazawa-u.ac.jp (M.S)
5 Department of Physics, Faculty of Science, Taif University, P.O. Box 11099, Taif 21944, Saudi Arabia; m.algethami@tu.edu.sa (M.A.); s.saeed@tu.edu.sa (S.S.A.)
6 Institute of Sustainable Energy, Universiti Tenaga Nasional (The National Energy University), Jalan IKRAM-UNITEN, 43000 Kajang, Malaysia; nowshad@uniten.edu.my
7 Graduate School of Pure and Applied Sciences, University of Tsukuba, Tsukuba, Ibaraki 305-8573, Japan
* Correspondence: akhtar@ukm.edu.my (M.A.); bashar@agni.com (M.S.B.)

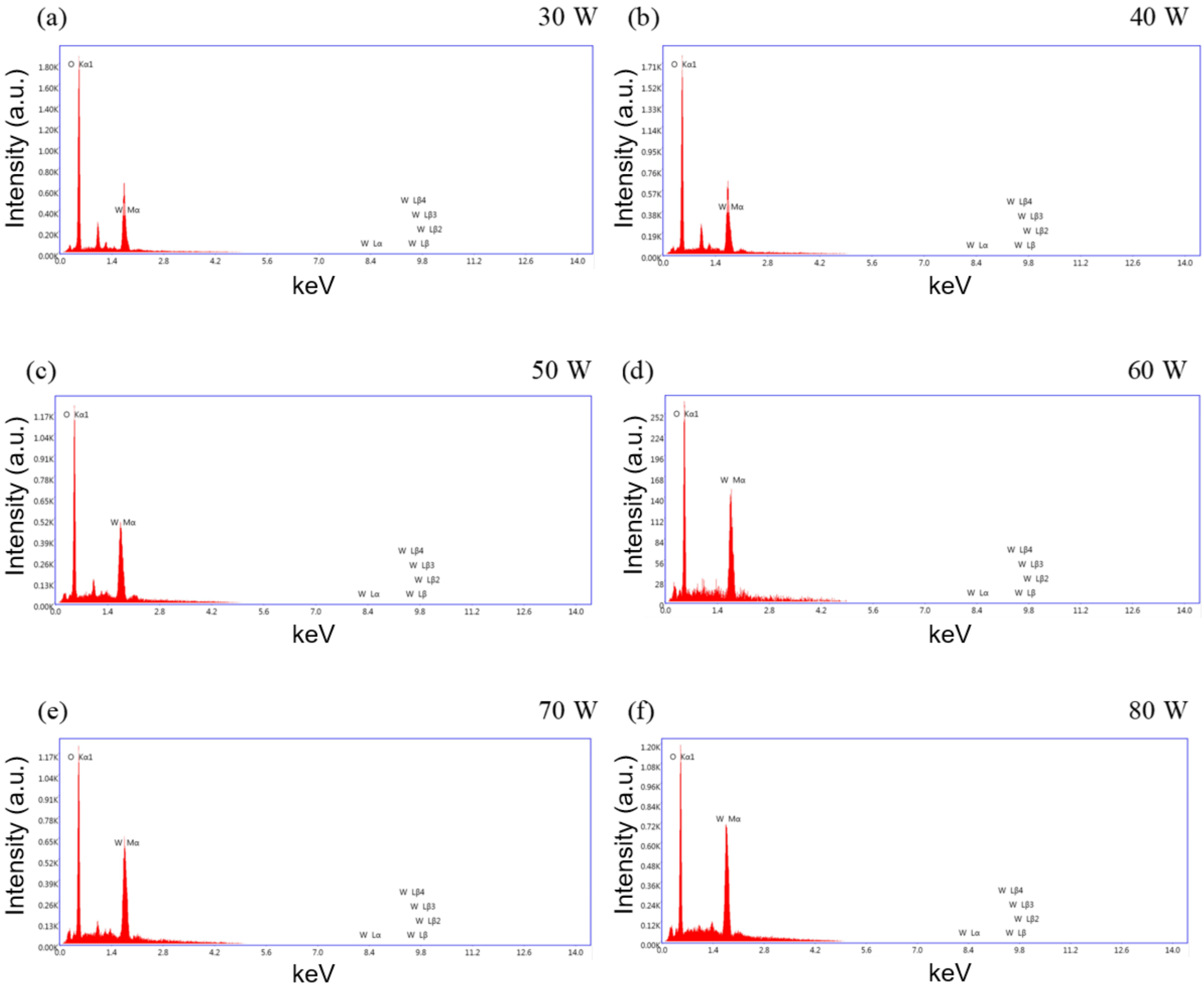

Figure S1. EDX images of the WO_x thin films, deposited at different sputtering powers:.(a) 30W, (b) 40 W, (c) 50 W, (d) 60 W, (e)70 W, (f) 80 W.

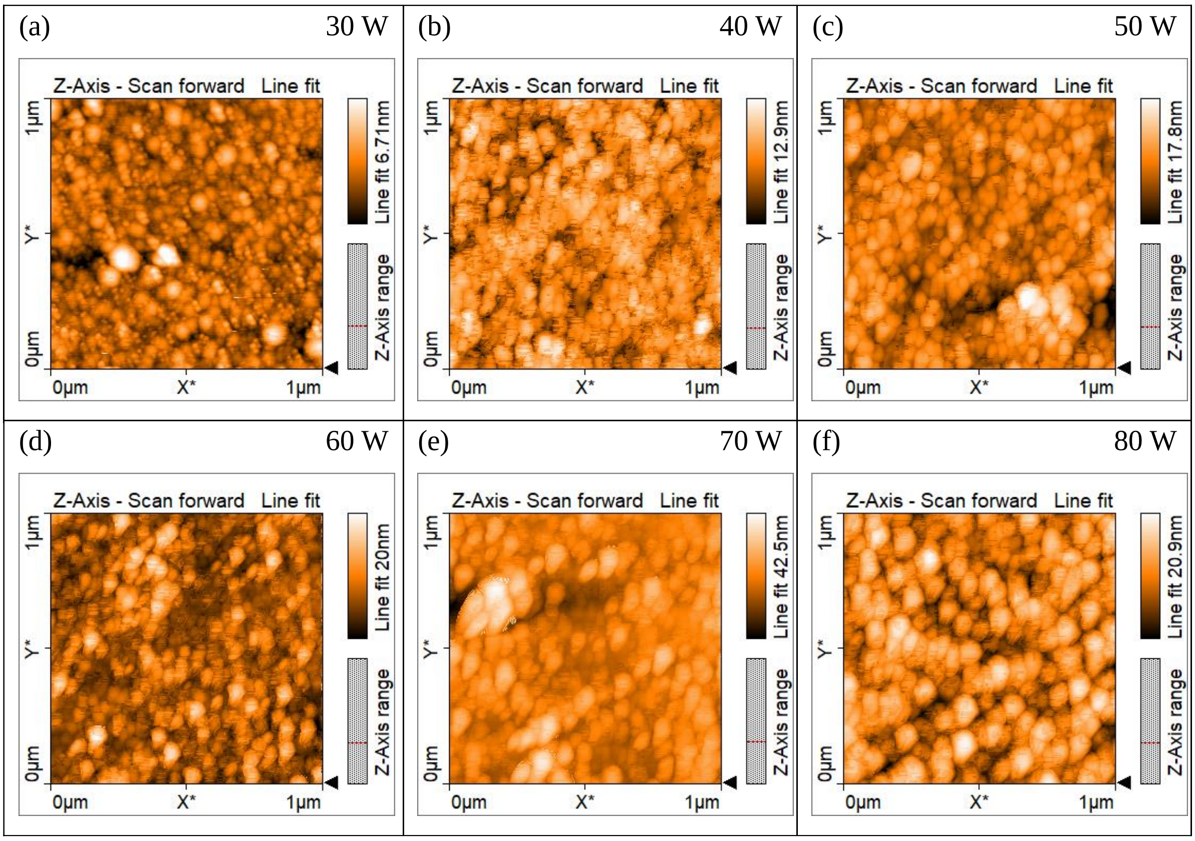

Figure S2. 2D AFM images of the WO_x thin films, deposited at different sputtering powers:.(a) 30W, (b) 40 W, (c) 50 W, (d) 60 W, (e)70 W, (f) 80 W.
